# Aqua­carbon­yl(ferrocenyldi­thio­phos­phon­ato-κ^2^ *S*,*S*′)bis­(tri­phenyl­phosphane-κ*P*)ruthenium(II) dichloromethane mono­solvate

**DOI:** 10.1107/S1600536813014311

**Published:** 2013-05-31

**Authors:** Hang Zhu, Qing Ma, Hua-Tian Shi, Qun Chen, Qian-Feng Zhang

**Affiliations:** a Department of Applied Chemistry, School of Petrochemical Engineering, Changzhou University, Jiangsu 213164, People’s Republic of China; b Institute of Molecular Engineering and Applied Chemistry, Anhui University of Technology, Ma’anshan, Anhui 243002, People’s Republic of China

## Abstract

The structure of the title complex, [FeRu(C_5_H_5_)(C_5_H_4_OPS_2_)(CO)(C_18_H_15_P)_2_(H_2_O)]·CH_2_Cl_2_, consists of one neutral [{FcP(O)S_2_}Ru(CO)(H_2_O)(PPh_3_)_2_] complex [Fc = Fe(η^5^-C_5_H_4_)(η^5^-C_5_H_5_)] and one CH_2_Cl_2_ solvent mol­ecule. The geometry around the Ru^II^ atom is pseudo-octa­hedral, with two *cis*-binding PPh_3_ ligands and one chelating bidentate [Fc(O)PS_2_]^2−^ ligand *via* two S atoms. The average Ru—S and Ru—P bond lengths are 2.434 (1) and 2.398 (1) Å, and the Ru—O and Ru—C bond lengths are 2.157 (3) and 1.826 (4) Å, respectively. In the crystal, pairs of O—H⋯O hydrogen bonds link adjacent mol­ecules into dimers.

## Related literature

For background to ferrocen­yl–phosphono­dithiol­ato com­plexes, see: Foreman *et al.* (1996 ▶); Gray *et al.* (2003 ▶, 2004 ▶); Haiduc (2001 ▶); Thomas *et al.* (2001 ▶); Van Zyl (2010 ▶). For a related structure, see: Liu *et al.* (2005 ▶); Wang *et al.* (2010 ▶); Zhang *et al.* (2001 ▶). For a description of the Cambridge Structural Database, see: Allen (2002 ▶).
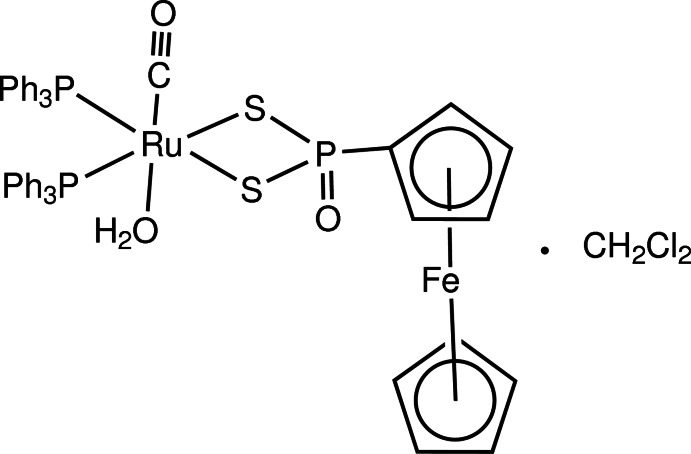


## Experimental

### 

#### Crystal data

[FeRu(C_5_H_5_)(C_5_H_4_OPS_2_)(CO)(C_18_H_15_P)_2_(H_2_O)]·CH_2_Cl_2_
*M* *_r_* = 1052.67
Triclinic, 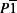

*a* = 12.1493 (9) Å
*b* = 14.2208 (11) Å
*c* = 14.7100 (11) Å
α = 101.811 (1)°
β = 98.278 (1)°
γ = 109.759 (1)°
*V* = 2277.7 (3) Å^3^
*Z* = 2
Mo *K*α radiation
μ = 1.01 mm^−1^
*T* = 296 K
0.24 × 0.15 × 0.08 mm

#### Data collection

Bruker SMART APEXII CCD area-detector diffractometer
Absorption correction: multi-scan (*SADABS*; Sheldrick, 1997 ▶) *T*_min_ = 0.794, *T*_max_ = 0.924
32262 measured reflections
10536 independent reflections
8221 reflections with *I* > 2σ(*I*)
*R*_int_ = 0.039

#### Refinement

*R*[*F* ^2^ > 2σ(*F* ^2^)] = 0.054
*wR*(*F* ^2^) = 0.154
*S* = 1.05
10536 reflections
549 parameters
2 restraints
H atoms treated by a mixture of independent and constrained refinement
Δρ_max_ = 3.98 e Å^−3^
Δρ_min_ = −0.67 e Å^−3^

### 

Data collection: *APEX2* (Bruker, 2005 ▶); cell refinement: *SAINT* (Bruker, 2005 ▶); data reduction: *SAINT*; program(s) used to solve structure: *SHELXS97* (Sheldrick, 2008 ▶); program(s) used to refine structure: *SHELXL97* (Sheldrick, 2008 ▶); molecular graphics: *SHELXTL* (Sheldrick, 2008 ▶); software used to prepare material for publication: *SHELXTL*.

## Supplementary Material

Crystal structure: contains datablock(s) I, global. DOI: 10.1107/S1600536813014311/ds2231sup1.cif

Structure factors: contains datablock(s) I. DOI: 10.1107/S1600536813014311/ds2231Isup2.hkl

Additional supplementary materials:  crystallographic information; 3D view; checkCIF report

## Figures and Tables

**Table 1:** Hydrogen-bond geometry (Å, °)

*D*—H⋯*A*	*D*—H	H⋯*A*	*D*⋯*A*	*D*—H⋯*A*
O3—H1*S*⋯O1^i^	0.82 (1)	1.72 (2)	2.515 (4)	162 (4)
O3—H2*S*⋯O3^i^	0.82 (1)	2.52 (6)	2.980 (6)	117 (5)

Symmetry code: (i) 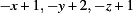.

**Fig. 1.**
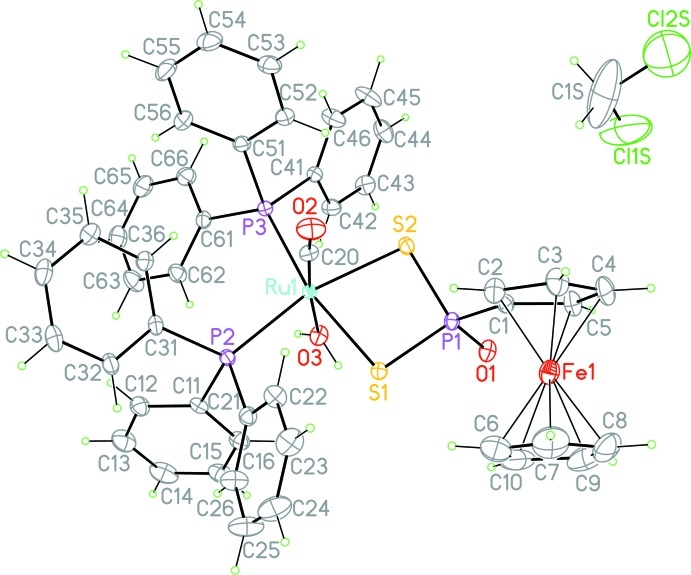
The structure of the title complex, showing the atom-numbering scheme and displacement ellipsoids at the 50% probability level.

**Fig. 2.**
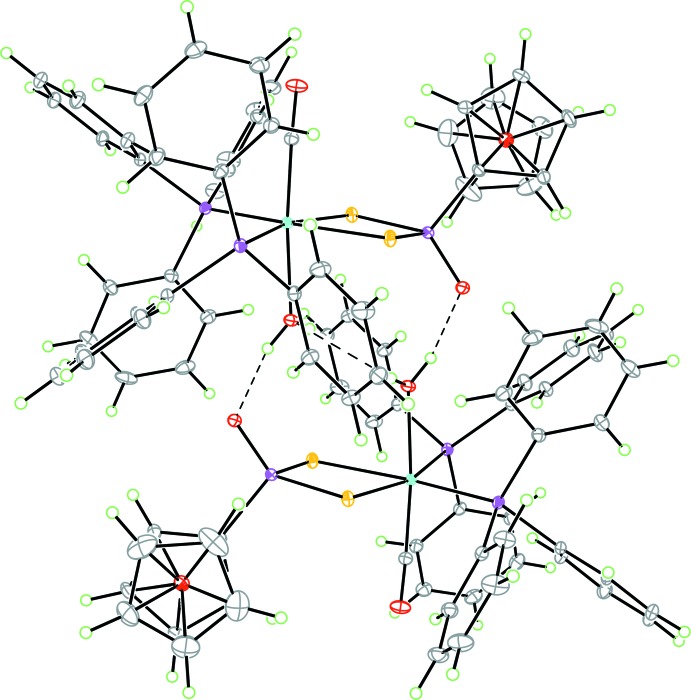
Two complex molecules are connected by intermolecular O—H···O(P) hydrogen bonds (dashed lines), forming a dimeric arrangement.

## supplementary crystallographic information

### Comment

Lawesson's Reagent (LR) [(*p*-MeO-C_6_H_4_)P(S)(µ-S)]_2_ was initially used for the purpose of a sulfur transfer reagent, especially to convert ketones to thiones, but was later used to form dithiophosphonic acids as well (Haiduc, 2001; Van Zyl, 2010). LR is formed through the reaction between P_4_S_10_ and anisole. Recognizing anisole to be an electron-rich aromatic, Woollins and co-workers skillfully introduced ferrocene, which performs similar electrophilic substitution type chemistry to afford the ferrocenyl derivative [FcP(S)(µ-S)]_2_ (Fc = Fe(η^5^-C_5_H_4_)(η^5^-C_5_H_5_)) (Gray *et al.*, 2004). This chemistry has been extended further by forming interesting ferrocenyl-type heterocycles. Similarly, [FcP(S)(µ-S)]_2_ can undergo a ring opening reaction by nucleophilic attack under suitable conditions, resulting in formation of the typical ferrocenyl-dithiophosphonate ligands, which may directly react with a range of metal ions to produce new heterometallic complexes containing the electron-rich and aromatic ferrocene groups (Gray *et al.*, 2003; Thomas *et al.*, 2001). As a part of research interest to the later transition metal-sulfur chemistry, we have recently reported ruthenium complexes with ferrocenyl-phosphonodithiolate as a dithio ligand (Wang *et al.*, 2010). We here describe the crystal structure of a ruthenium(II)-ferrocenyl-dithiophosphonato complex [{FcP(O)S_2_}Ru(CO)(H_2_O)(PPh_3)_2].CH_2Cl_2 (Fc = Fe(η^5^-C_5_H_4_)(η^5^-C_5_H_5_)) in this paper.

The title complex crystallizes in triclinic space group *P*-1 with two molecules in the unit cell, as shown in Fig. 1. The ruthenium center has an octahedral coordination environment with the H_2_O and CO ligands mutually *trans*. The [FcP(O)S_2_]^2-^ acts as a chelating ligand through its two sulfur atoms to bond the ruthenium center with the bite angle S(1)—Ru(1)—S(2) of 79.74 (4)° which agrees with those in *cis*-[Ru(CO){FcP(OCH_3_)PS_2_}_2_(PPh_3_)] [78.43 (3)° and 79.84 (3)°] (Wang *et al.*, 2010). Two *cis* PPh_3_ ligands bind to the ruthenium center with the P—Ru—P angle of 106.97 (4)°, and one chelating [FcP(O)S_2_]^2-^ ligands form the basal plane. The average Ru—S bond length (av. 2.4337 (20) Å) in the title complex is compatible to that in *cis*-[Ru(CO){FcP(OCH_3_)PS_2_}_2_(PPh_3_)] (av. 2.4854 (11) Å) (Wang *et al.*, 2010). The Ru—O bond length of 2.159 (3) Å is similar to that observed for *trans*-Ru[N(Ph_2_PS)_2_]_2_(H_2_O)(NH_3_) (2.118 (4) Å) (Zhang *et al.*, 2001). The Ru—C bond length and Ru—C—O bond angle in the title complex are 1.826 (4) Å and 176.9 (4)°, respectively, which are comparable to those in [Ru(CO){*ARP*(O)S_2_}(PPh_3_)_2_]_2_ (Ar = *p*-C_6_H_4_OMe) (Ru—C = 1.804 (4) Å and Ru—C—O = 174.8 (4)°) (Wang *et al.*, 2010) and [RuH(CO){S_2_P(OEt)_2_}(PPh_3_)_2_] (Ru—C = 1.829 (4) Å and Ru—C—O = 175.4 (4)°) (Liu *et al.* 2005). A pair of head-to-tail intermolecular O—H···Oa (a: *x* + 1, *y* + 2, *z* + 1) hydrogen bonds (O3—H1S···O1a: 1.719 (16) Å, 2.516 (4) Å, 162 (4)°; O3—H2S···O3a: 2.53 (6) Å, 2.981 (6) Å, 116 (5)°) linking adjacent molecules to form a dimmer was observed in the crystal packing.

### Experimental

To a slurry of [FcP(S)(µ-S)]_2_ (56 mg, 0.10 mmol) and 17% NH_3_.H_2_O (0.2 ml) in THF (10 ml) was added the grey solid [RuHCl(CO)(PPh_3_)_3_] (188 mg, 0.20 mmol). The mixture was stirred at room temperature overnight and the brown solution was obtained. The solvent was removed *in vacuo* and the residue was recrystallized from CH_2_Cl_2_/hexane to give yellow crystalline solids of [{FcP(O)S_2_}Ru(CO)(H_2_O)(PPh_3_)_2_].CH_2_Cl_2_ in five days at room temperature. Yield: 68 mg, 0.065 mmol, 32% (based on Ru). Anal. Calcd. for C_47_H_41_O_3_P_3_S_2_FeRu.(CH_2_Cl_2_): C, 54.76; H, 4.12%. Found: C, 54.72; H, 4.08%.

### Refinement

The structure was solved by direct methods and refined by full-matrix least-squares procedure based on F^2^. All C Hydrogen atoms were placed in geometrically idealized positions and refined isotropically with a riding model for C-*sp*^2^ [C—H = 0.93 Å and with *U*_iso_(H) = 1.2*U*_eq_(C)] and C-*sp*^3^ [C—H = 0.97 Å and with *U*_iso_(H) = 1.5*U*_eq_(C)].

### Crystal data

**Table:**

[FeRu(C_5_H_5_)(C_5_H_4_OPS_2_)(CO)(C_18_H_15_P)_2_(H_2_O)]·CH_2_Cl_2_	*Z* = 2
*M**_r_* = 1052.67	*F*(000) = 1072
Triclinic, *P*1	*D*_x_ = 1.535 Mg m^−^^3^
Hall symbol: -P 1	Mo *K*α radiation, λ = 0.71073 Å
*a* = 12.1493 (9) Å	Cell parameters from 6761 reflections
*b* = 14.2208 (11) Å	θ = 2.4–25.7°
*c* = 14.7100 (11) Å	µ = 1.01 mm^−^^1^
α = 101.811 (1)°	*T* = 296 K
β = 98.278 (1)°	Block, yellow
γ = 109.759 (1)°	0.24 × 0.15 × 0.08 mm
*V* = 2277.7 (3) Å^3^	

### Data collection

**Table:**

Bruker SMART APEXII CCD area-detector diffractometer	10536 independent reflections
Radiation source: fine-focus sealed tube	8221 reflections with *I* > 2σ(*I*)
Graphite monochromator	*R*_int_ = 0.039
phi and ω scans	θ_max_ = 27.6°, θ_min_ = 2.4°
Absorption correction: multi-scan (*SADABS*; Sheldrick, 1997)	*h* = −15→15
*T*_min_ = 0.794, *T*_max_ = 0.924	*k* = −18→18
32262 measured reflections	*l* = −19→19

### Refinement

**Table:**

Refinement on *F*^2^	Primary atom site location: structure-invariant direct methods
Least-squares matrix: full	Secondary atom site location: difference Fourier map
*R*[*F*^2^ > 2σ(*F*^2^)] = 0.054	Hydrogen site location: inferred from neighbouring sites
*wR*(*F*^2^) = 0.154	H atoms treated by a mixture of independent and constrained refinement
*S* = 1.05	*w* = 1/[σ^2^(*F*_o_^2^) + (0.0853*P*)^2^ + 2.0442*P*] where *P* = (*F*_o_^2^ + 2*F*_c_^2^)/3
10536 reflections	(Δ/σ)_max_ = 0.001
549 parameters	Δρ_max_ = 3.98 e Å^−^^3^
2 restraints	Δρ_min_ = −0.67 e Å^−^^3^

### Special details

**Table:**

Geometry. All e.s.d.'s (except the e.s.d. in the dihedral angle between two l.s. planes) are estimated using the full covariance matrix. The cell e.s.d.'s are taken into account individually in the estimation of e.s.d.'s in distances, angles and torsion angles; correlations between e.s.d.'s in cell parameters are only used when they are defined by crystal symmetry. An approximate (isotropic) treatment of cell e.s.d.'s is used for estimating e.s.d.'s involving l.s. planes.
Refinement. Refinement of *F*^2^ against ALL reflections. The weighted *R*-factor *wR* and goodness of fit *S* are based on *F*^2^, conventional *R*-factors *R* are based on *F*, with *F* set to zero for negative *F*^2^. The threshold expression of *F*^2^ > σ(*F*^2^) is used only for calculating *R*-factors(gt) *etc*. and is not relevant to the choice of reflections for refinement. *R*-factors based on *F*^2^ are statistically about twice as large as those based on *F*, and *R*- factors based on ALL data will be even larger.

### Fractional atomic coordinates and isotropic or equivalent isotropic displacement parameters (Å^2^)

**Table:**

	*x*	*y*	*z*	*U*_iso_*/*U*_eq_	
Ru1	0.57682 (3)	0.84933 (2)	0.60821 (2)	0.02666 (10)	
Fe1	0.34922 (7)	0.55279 (5)	0.22580 (5)	0.04881 (18)	
O3	0.5232 (3)	0.9764 (2)	0.5934 (2)	0.0406 (7)	
H1S	0.541 (4)	1.0333 (16)	0.632 (2)	0.029 (11)*	
H2S	0.460 (3)	0.962 (5)	0.555 (4)	0.09 (2)*	
S1	0.40195 (9)	0.74574 (8)	0.47898 (7)	0.0361 (2)	
S2	0.66350 (9)	0.87473 (8)	0.47088 (7)	0.0378 (2)	
P1	0.49822 (9)	0.78762 (7)	0.38051 (7)	0.0305 (2)	
P2	0.44859 (9)	0.80208 (7)	0.71339 (7)	0.0299 (2)	
P3	0.76866 (9)	0.97232 (8)	0.70618 (7)	0.0306 (2)	
O1	0.4530 (3)	0.8442 (2)	0.3179 (2)	0.0418 (7)	
O2	0.6390 (3)	0.6647 (2)	0.6043 (3)	0.0588 (9)	
C1	0.5005 (4)	0.6752 (3)	0.3034 (3)	0.0354 (8)	
C2	0.5009 (5)	0.5821 (4)	0.3236 (3)	0.0514 (12)	
H2	0.5042	0.5688	0.3831	0.062*	
C3	0.4953 (5)	0.5126 (4)	0.2371 (4)	0.0593 (13)	
H3	0.4951	0.4461	0.2304	0.071*	
C4	0.4899 (5)	0.5611 (4)	0.1627 (4)	0.0629 (14)	
H4	0.4848	0.5321	0.0988	0.075*	
C5	0.4937 (5)	0.6615 (3)	0.2028 (3)	0.0495 (11)	
H5	0.4920	0.7104	0.1698	0.059*	
C6	0.2098 (7)	0.5002 (9)	0.2869 (7)	0.109 (3)	
H6	0.2179	0.4986	0.3503	0.131*	
C7	0.2044 (7)	0.4244 (6)	0.2133 (8)	0.107 (3)	
H7	0.2062	0.3607	0.2183	0.128*	
C8	0.1960 (6)	0.4522 (6)	0.1304 (6)	0.093 (2)	
H8	0.1915	0.4117	0.0706	0.111*	
C9	0.1952 (7)	0.5517 (8)	0.1503 (8)	0.114 (3)	
H9	0.1916	0.5910	0.1074	0.136*	
C10	0.2012 (6)	0.5817 (7)	0.2517 (8)	0.112 (3)	
H10	0.1995	0.6435	0.2865	0.135*	
C11	0.3794 (3)	0.8935 (3)	0.7572 (3)	0.0352 (8)	
C12	0.3908 (4)	0.9360 (3)	0.8534 (3)	0.0473 (10)	
H12	0.4339	0.9171	0.8991	0.057*	
C13	0.3380 (5)	1.0067 (4)	0.8819 (4)	0.0642 (14)	
H13	0.3455	1.0344	0.9466	0.077*	
C14	0.2758 (5)	1.0353 (4)	0.8160 (5)	0.0689 (17)	
H14	0.2415	1.0832	0.8356	0.083*	
C15	0.2630 (5)	0.9939 (4)	0.7202 (5)	0.0608 (14)	
H15	0.2199	1.0137	0.6753	0.073*	
C16	0.3142 (4)	0.9226 (4)	0.6904 (4)	0.0479 (11)	
H16	0.3048	0.8942	0.6255	0.058*	
C20	0.6172 (3)	0.7368 (3)	0.6083 (3)	0.0349 (8)	
C21	0.3166 (4)	0.6803 (3)	0.6583 (3)	0.0378 (9)	
C22	0.3325 (4)	0.5913 (3)	0.6148 (3)	0.0485 (11)	
H22	0.4094	0.5950	0.6103	0.058*	
C23	0.2363 (5)	0.4970 (4)	0.5777 (4)	0.0632 (14)	
H23	0.2485	0.4380	0.5483	0.076*	
C24	0.1235 (5)	0.4914 (4)	0.5847 (5)	0.0755 (18)	
H24	0.0585	0.4284	0.5593	0.091*	
C25	0.1053 (5)	0.5774 (5)	0.6287 (5)	0.0790 (19)	
H25	0.0285	0.5722	0.6349	0.095*	
C26	0.2008 (4)	0.6723 (4)	0.6642 (4)	0.0592 (13)	
H26	0.1874	0.7311	0.6922	0.071*	
C31	0.5046 (4)	0.7699 (3)	0.8209 (3)	0.0344 (8)	
C32	0.4262 (4)	0.7171 (3)	0.8702 (3)	0.0430 (10)	
H32	0.3440	0.6993	0.8496	0.052*	
C33	0.4688 (5)	0.6907 (4)	0.9492 (3)	0.0532 (12)	
H33	0.4154	0.6559	0.9818	0.064*	
C34	0.5900 (5)	0.7156 (4)	0.9800 (3)	0.0547 (12)	
H34	0.6187	0.6975	1.0332	0.066*	
C35	0.6690 (5)	0.7675 (4)	0.9317 (3)	0.0531 (12)	
H35	0.7511	0.7843	0.9522	0.064*	
C36	0.6264 (4)	0.7946 (3)	0.8530 (3)	0.0420 (9)	
H36	0.6803	0.8300	0.8210	0.050*	
C41	0.8587 (3)	1.0592 (3)	0.6437 (3)	0.0332 (8)	
C42	0.8153 (4)	1.1299 (3)	0.6144 (3)	0.0447 (10)	
H42	0.7427	1.1315	0.6262	0.054*	
C43	0.8791 (4)	1.1975 (3)	0.5681 (4)	0.0500 (11)	
H43	0.8490	1.2441	0.5485	0.060*	
C44	0.9874 (5)	1.1962 (4)	0.5508 (4)	0.0587 (13)	
H44	1.0303	1.2416	0.5194	0.070*	
C45	1.0315 (5)	1.1275 (4)	0.5800 (5)	0.0682 (16)	
H45	1.1048	1.1270	0.5691	0.082*	
C46	0.9668 (5)	1.0587 (4)	0.6261 (4)	0.0563 (13)	
H46	0.9969	1.0120	0.6452	0.068*	
C51	0.8706 (3)	0.9121 (3)	0.7488 (3)	0.0367 (9)	
C52	0.8946 (4)	0.8419 (3)	0.6820 (3)	0.0413 (9)	
H52	0.8600	0.8274	0.6175	0.050*	
C53	0.9693 (4)	0.7934 (4)	0.7102 (4)	0.0519 (12)	
H53	0.9858	0.7475	0.6646	0.062*	
C54	1.0187 (5)	0.8123 (4)	0.8038 (4)	0.0593 (13)	
H54	1.0689	0.7793	0.8223	0.071*	
C55	0.9948 (5)	0.8805 (4)	0.8720 (4)	0.0607 (14)	
H55	1.0275	0.8923	0.9364	0.073*	
C56	0.9218 (4)	0.9312 (4)	0.8444 (3)	0.0490 (11)	
H56	0.9072	0.9782	0.8902	0.059*	
C61	0.7790 (4)	1.0714 (3)	0.8121 (3)	0.0356 (8)	
C62	0.6762 (4)	1.0715 (3)	0.8422 (3)	0.0453 (10)	
H62	0.6019	1.0203	0.8094	0.054*	
C63	0.6843 (5)	1.1488 (4)	0.9222 (4)	0.0610 (14)	
H63	0.6154	1.1477	0.9432	0.073*	
C64	0.7919 (5)	1.2250 (4)	0.9693 (4)	0.0605 (14)	
H64	0.7963	1.2762	1.0219	0.073*	
C65	0.8950 (5)	1.2269 (4)	0.9395 (4)	0.0604 (13)	
H65	0.9688	1.2789	0.9725	0.072*	
C66	0.8888 (4)	1.1513 (4)	0.8603 (3)	0.0508 (11)	
H66	0.9581	1.1540	0.8392	0.061*	
C1S	0.8456 (18)	0.6112 (13)	0.2091 (9)	0.264 (11)	
H1S1	0.7820	0.5791	0.2390	0.317*	
H1S2	0.9174	0.6505	0.2597	0.317*	
Cl1S	0.8099 (3)	0.6917 (4)	0.1639 (4)	0.2144 (19)	
Cl2S	0.8735 (4)	0.5084 (4)	0.1346 (4)	0.2133 (17)	

### Atomic displacement parameters (Å^2^)

**Table:**

	*U*^11^	*U*^22^	*U*^33^	*U*^12^	*U*^13^	*U*^23^
Ru1	0.02830 (16)	0.02926 (16)	0.02479 (16)	0.01314 (12)	0.00753 (12)	0.00756 (11)
Fe1	0.0577 (4)	0.0377 (3)	0.0416 (4)	0.0124 (3)	0.0055 (3)	0.0049 (3)
O3	0.0535 (19)	0.0318 (15)	0.0399 (17)	0.0233 (14)	0.0068 (15)	0.0077 (13)
S1	0.0323 (5)	0.0421 (5)	0.0291 (5)	0.0102 (4)	0.0071 (4)	0.0061 (4)
S2	0.0344 (5)	0.0441 (5)	0.0301 (5)	0.0086 (4)	0.0113 (4)	0.0077 (4)
P1	0.0372 (5)	0.0279 (4)	0.0271 (5)	0.0140 (4)	0.0073 (4)	0.0060 (4)
P2	0.0315 (5)	0.0345 (5)	0.0289 (5)	0.0156 (4)	0.0109 (4)	0.0116 (4)
P3	0.0287 (5)	0.0366 (5)	0.0280 (5)	0.0145 (4)	0.0065 (4)	0.0087 (4)
O1	0.0580 (19)	0.0330 (14)	0.0340 (15)	0.0203 (13)	0.0051 (13)	0.0071 (12)
O2	0.055 (2)	0.0425 (17)	0.087 (3)	0.0301 (16)	0.0137 (19)	0.0167 (17)
C1	0.040 (2)	0.0319 (19)	0.033 (2)	0.0151 (16)	0.0078 (17)	0.0031 (15)
C2	0.072 (3)	0.048 (2)	0.046 (3)	0.037 (2)	0.012 (2)	0.015 (2)
C3	0.083 (4)	0.042 (2)	0.058 (3)	0.035 (3)	0.018 (3)	0.004 (2)
C4	0.087 (4)	0.048 (3)	0.048 (3)	0.024 (3)	0.029 (3)	−0.002 (2)
C5	0.067 (3)	0.041 (2)	0.039 (2)	0.018 (2)	0.021 (2)	0.0084 (19)
C6	0.061 (4)	0.141 (8)	0.102 (7)	0.005 (5)	0.030 (4)	0.033 (6)
C7	0.084 (5)	0.066 (4)	0.134 (8)	−0.008 (4)	0.017 (5)	0.021 (5)
C8	0.077 (5)	0.082 (5)	0.078 (5)	0.016 (4)	−0.013 (4)	−0.019 (4)
C9	0.073 (5)	0.122 (7)	0.142 (8)	0.034 (5)	−0.019 (5)	0.065 (6)
C10	0.044 (3)	0.105 (6)	0.143 (8)	0.024 (4)	0.002 (4)	−0.038 (6)
C11	0.0313 (19)	0.0363 (19)	0.043 (2)	0.0152 (16)	0.0177 (17)	0.0122 (17)
C12	0.049 (3)	0.049 (2)	0.046 (3)	0.023 (2)	0.016 (2)	0.006 (2)
C13	0.067 (3)	0.055 (3)	0.073 (4)	0.032 (3)	0.027 (3)	0.001 (3)
C14	0.065 (3)	0.047 (3)	0.114 (5)	0.037 (3)	0.042 (4)	0.021 (3)
C15	0.054 (3)	0.067 (3)	0.092 (4)	0.040 (3)	0.034 (3)	0.046 (3)
C16	0.046 (3)	0.055 (3)	0.057 (3)	0.027 (2)	0.022 (2)	0.027 (2)
C20	0.0305 (19)	0.0347 (19)	0.038 (2)	0.0132 (16)	0.0042 (17)	0.0090 (16)
C21	0.039 (2)	0.039 (2)	0.034 (2)	0.0123 (17)	0.0093 (17)	0.0130 (17)
C22	0.051 (3)	0.042 (2)	0.053 (3)	0.017 (2)	0.016 (2)	0.013 (2)
C23	0.071 (4)	0.034 (2)	0.070 (4)	0.011 (2)	0.006 (3)	0.007 (2)
C24	0.056 (3)	0.044 (3)	0.102 (5)	0.001 (2)	−0.006 (3)	0.014 (3)
C25	0.038 (3)	0.059 (3)	0.125 (6)	0.009 (2)	0.009 (3)	0.017 (4)
C26	0.043 (3)	0.049 (3)	0.083 (4)	0.016 (2)	0.012 (3)	0.014 (3)
C31	0.045 (2)	0.0371 (19)	0.0268 (19)	0.0196 (17)	0.0129 (17)	0.0112 (15)
C32	0.051 (3)	0.053 (2)	0.034 (2)	0.023 (2)	0.020 (2)	0.0179 (19)
C33	0.076 (4)	0.054 (3)	0.041 (3)	0.028 (3)	0.028 (2)	0.024 (2)
C34	0.078 (4)	0.060 (3)	0.036 (2)	0.034 (3)	0.011 (2)	0.021 (2)
C35	0.054 (3)	0.066 (3)	0.045 (3)	0.028 (2)	0.007 (2)	0.022 (2)
C36	0.043 (2)	0.051 (2)	0.036 (2)	0.0176 (19)	0.0098 (19)	0.0195 (19)
C41	0.0304 (19)	0.0360 (19)	0.032 (2)	0.0111 (15)	0.0083 (16)	0.0085 (16)
C42	0.039 (2)	0.048 (2)	0.054 (3)	0.0187 (19)	0.013 (2)	0.021 (2)
C43	0.053 (3)	0.042 (2)	0.058 (3)	0.017 (2)	0.013 (2)	0.021 (2)
C44	0.074 (4)	0.047 (3)	0.063 (3)	0.018 (2)	0.037 (3)	0.023 (2)
C45	0.065 (3)	0.066 (3)	0.106 (5)	0.037 (3)	0.058 (3)	0.042 (3)
C46	0.054 (3)	0.056 (3)	0.082 (4)	0.032 (2)	0.036 (3)	0.036 (3)
C51	0.032 (2)	0.045 (2)	0.040 (2)	0.0177 (17)	0.0098 (17)	0.0185 (18)
C52	0.038 (2)	0.049 (2)	0.042 (2)	0.0217 (19)	0.0088 (19)	0.0153 (19)
C53	0.045 (3)	0.058 (3)	0.066 (3)	0.031 (2)	0.019 (2)	0.023 (2)
C54	0.050 (3)	0.069 (3)	0.075 (4)	0.035 (3)	0.012 (3)	0.036 (3)
C55	0.055 (3)	0.079 (4)	0.050 (3)	0.028 (3)	−0.002 (2)	0.029 (3)
C56	0.049 (3)	0.062 (3)	0.041 (2)	0.027 (2)	0.005 (2)	0.018 (2)
C61	0.042 (2)	0.038 (2)	0.0287 (19)	0.0179 (17)	0.0078 (17)	0.0077 (16)
C62	0.046 (2)	0.044 (2)	0.043 (2)	0.0159 (19)	0.015 (2)	0.0040 (19)
C63	0.067 (3)	0.059 (3)	0.057 (3)	0.024 (3)	0.031 (3)	0.002 (2)
C64	0.084 (4)	0.053 (3)	0.041 (3)	0.029 (3)	0.012 (3)	0.001 (2)
C65	0.059 (3)	0.053 (3)	0.048 (3)	0.010 (2)	−0.004 (2)	0.000 (2)
C66	0.041 (2)	0.052 (3)	0.046 (3)	0.011 (2)	0.004 (2)	0.000 (2)
C1S	0.42 (3)	0.192 (14)	0.090 (9)	0.012 (16)	−0.011 (12)	0.071 (10)
Cl1S	0.0880 (19)	0.266 (5)	0.281 (5)	0.041 (2)	0.017 (2)	0.122 (4)
Cl2S	0.175 (3)	0.190 (4)	0.221 (4)	0.019 (3)	0.041 (3)	0.034 (3)

### Geometric parameters (Å, º)

**Table:**

Ru1—C20	1.826 (4)	C21—C22	1.385 (6)
Ru1—O3	2.157 (3)	C21—C26	1.389 (6)
Ru1—P2	2.3842 (10)	C22—C23	1.385 (7)
Ru1—P3	2.4110 (10)	C22—H22	0.9300
Ru1—S1	2.4232 (10)	C23—C24	1.366 (8)
Ru1—S2	2.4443 (10)	C23—H23	0.9300
Fe1—C7	2.015 (7)	C24—C25	1.365 (8)
Fe1—C6	2.023 (7)	C24—H24	0.9300
Fe1—C9	2.027 (7)	C25—C26	1.386 (7)
Fe1—C8	2.027 (6)	C25—H25	0.9300
Fe1—C2	2.028 (5)	C26—H26	0.9300
Fe1—C1	2.033 (4)	C31—C36	1.384 (6)
Fe1—C5	2.033 (5)	C31—C32	1.392 (6)
Fe1—C3	2.035 (5)	C32—C33	1.376 (6)
Fe1—C4	2.040 (5)	C32—H32	0.9300
Fe1—C10	2.045 (7)	C33—C34	1.374 (8)
O3—H1S	0.824 (10)	C33—H33	0.9300
O3—H2S	0.818 (10)	C34—C35	1.380 (7)
S1—P1	2.0472 (14)	C34—H34	0.9300
S2—P1	2.0517 (14)	C35—C36	1.379 (6)
P1—O1	1.502 (3)	C35—H35	0.9300
P1—C1	1.772 (4)	C36—H36	0.9300
P2—C11	1.834 (4)	C41—C46	1.376 (6)
P2—C31	1.836 (4)	C41—C42	1.394 (5)
P2—C21	1.844 (4)	C42—C43	1.381 (6)
P3—C61	1.831 (4)	C42—H42	0.9300
P3—C51	1.837 (4)	C43—C44	1.381 (7)
P3—C41	1.846 (4)	C43—H43	0.9300
O2—C20	1.136 (5)	C44—C45	1.373 (7)
C1—C2	1.417 (6)	C44—H44	0.9300
C1—C5	1.439 (6)	C45—C46	1.393 (7)
C2—C3	1.421 (6)	C45—H45	0.9300
C2—H2	0.9300	C46—H46	0.9300
C3—C4	1.412 (7)	C51—C56	1.384 (6)
C3—H3	0.9300	C51—C52	1.388 (6)
C4—C5	1.410 (6)	C52—C53	1.379 (6)
C4—H4	0.9300	C52—H52	0.9300
C5—H5	0.9300	C53—C54	1.354 (7)
C6—C7	1.339 (12)	C53—H53	0.9300
C6—C10	1.391 (12)	C54—C55	1.381 (8)
C6—H6	0.9300	C54—H54	0.9300
C7—C8	1.358 (11)	C55—C56	1.385 (6)
C7—H7	0.9300	C55—H55	0.9300
C8—C9	1.388 (11)	C56—H56	0.9300
C8—H8	0.9300	C61—C62	1.384 (6)
C9—C10	1.449 (12)	C61—C66	1.395 (6)
C9—H9	0.9300	C62—C63	1.403 (6)
C10—H10	0.9300	C62—H62	0.9300
C11—C12	1.387 (6)	C63—C64	1.355 (8)
C11—C16	1.388 (6)	C63—H63	0.9300
C12—C13	1.393 (6)	C64—C65	1.379 (8)
C12—H12	0.9300	C64—H64	0.9300
C13—C14	1.355 (8)	C65—C66	1.387 (7)
C13—H13	0.9300	C65—H65	0.9300
C14—C15	1.376 (8)	C66—H66	0.9300
C14—H14	0.9300	C1S—Cl1S	1.582 (16)
C15—C16	1.391 (6)	C1S—Cl2S	1.80 (2)
C15—H15	0.9300	C1S—H1S1	0.9700
C16—H16	0.9300	C1S—H1S2	0.9700
			
C20—Ru1—O3	174.49 (15)	C9—C8—H8	125.9
C20—Ru1—P2	90.26 (13)	Fe1—C8—H8	125.7
O3—Ru1—P2	92.66 (9)	C8—C9—C10	105.8 (8)
C20—Ru1—P3	94.08 (12)	C8—C9—Fe1	70.0 (4)
O3—Ru1—P3	89.54 (9)	C10—C9—Fe1	69.8 (4)
P2—Ru1—P3	106.97 (4)	C8—C9—H9	127.1
C20—Ru1—S1	91.03 (13)	C10—C9—H9	127.1
O3—Ru1—S1	84.48 (9)	Fe1—C9—H9	124.7
P2—Ru1—S1	86.51 (4)	C6—C10—C9	106.7 (7)
P3—Ru1—S1	165.53 (4)	C6—C10—Fe1	69.1 (4)
C20—Ru1—S2	90.95 (13)	C9—C10—Fe1	68.5 (4)
O3—Ru1—S2	85.10 (9)	C6—C10—H10	126.7
P2—Ru1—S2	166.20 (4)	C9—C10—H10	126.7
P3—Ru1—S2	86.65 (3)	Fe1—C10—H10	127.3
S1—Ru1—S2	79.73 (4)	C12—C11—C16	118.7 (4)
C7—Fe1—C6	38.7 (3)	C12—C11—P2	123.2 (3)
C7—Fe1—C9	66.7 (4)	C16—C11—P2	118.0 (3)
C6—Fe1—C9	68.5 (4)	C11—C12—C13	120.3 (5)
C7—Fe1—C8	39.3 (3)	C11—C12—H12	119.8
C6—Fe1—C8	66.7 (4)	C13—C12—H12	119.8
C9—Fe1—C8	40.0 (3)	C14—C13—C12	120.3 (5)
C7—Fe1—C2	118.0 (3)	C14—C13—H13	119.8
C6—Fe1—C2	106.7 (3)	C12—C13—H13	119.8
C9—Fe1—C2	166.5 (4)	C13—C14—C15	120.3 (5)
C8—Fe1—C2	151.0 (3)	C13—C14—H14	119.8
C7—Fe1—C1	152.2 (3)	C15—C14—H14	119.8
C6—Fe1—C1	119.6 (3)	C14—C15—C16	120.1 (5)
C9—Fe1—C1	129.5 (3)	C14—C15—H15	119.9
C8—Fe1—C1	167.3 (3)	C16—C15—H15	119.9
C2—Fe1—C1	40.84 (17)	C11—C16—C15	120.1 (5)
C7—Fe1—C5	164.7 (3)	C11—C16—H16	119.9
C6—Fe1—C5	155.7 (4)	C15—C16—H16	119.9
C9—Fe1—C5	109.9 (3)	O2—C20—Ru1	176.9 (4)
C8—Fe1—C5	128.6 (3)	C22—C21—C26	118.0 (4)
C2—Fe1—C5	69.0 (2)	C22—C21—P2	119.7 (3)
C1—Fe1—C5	41.46 (17)	C26—C21—P2	122.2 (3)
C7—Fe1—C3	107.4 (3)	C23—C22—C21	121.4 (5)
C6—Fe1—C3	125.1 (4)	C23—C22—H22	119.3
C9—Fe1—C3	152.2 (4)	C21—C22—H22	119.3
C8—Fe1—C3	117.9 (3)	C24—C23—C22	119.4 (5)
C2—Fe1—C3	40.93 (19)	C24—C23—H23	120.3
C1—Fe1—C3	68.67 (18)	C22—C23—H23	120.3
C5—Fe1—C3	68.2 (2)	C23—C24—C25	120.6 (5)
C7—Fe1—C4	126.8 (3)	C23—C24—H24	119.7
C6—Fe1—C4	162.2 (4)	C25—C24—H24	119.7
C9—Fe1—C4	119.5 (4)	C24—C25—C26	120.2 (5)
C8—Fe1—C4	108.2 (3)	C24—C25—H25	119.9
C2—Fe1—C4	69.0 (2)	C26—C25—H25	119.9
C1—Fe1—C4	69.13 (18)	C25—C26—C21	120.4 (5)
C5—Fe1—C4	40.51 (18)	C25—C26—H26	119.8
C3—Fe1—C4	40.6 (2)	C21—C26—H26	119.8
C7—Fe1—C10	65.9 (4)	C36—C31—C32	118.2 (4)
C6—Fe1—C10	40.0 (4)	C36—C31—P2	120.5 (3)
C9—Fe1—C10	41.7 (4)	C32—C31—P2	121.2 (3)
C8—Fe1—C10	67.5 (3)	C33—C32—C31	120.9 (5)
C2—Fe1—C10	126.7 (3)	C33—C32—H32	119.6
C1—Fe1—C10	109.5 (2)	C31—C32—H32	119.6
C5—Fe1—C10	122.4 (3)	C34—C33—C32	120.2 (5)
C3—Fe1—C10	163.0 (4)	C34—C33—H33	119.9
C4—Fe1—C10	156.0 (4)	C32—C33—H33	119.9
Ru1—O3—H1S	130 (3)	C33—C34—C35	119.8 (4)
Ru1—O3—H2S	116 (5)	C33—C34—H34	120.1
H1S—O3—H2S	110 (5)	C35—C34—H34	120.1
P1—S1—Ru1	90.85 (5)	C36—C35—C34	120.1 (5)
P1—S2—Ru1	90.15 (4)	C36—C35—H35	120.0
O1—P1—C1	106.63 (18)	C34—C35—H35	120.0
O1—P1—S1	116.18 (13)	C35—C36—C31	120.9 (4)
C1—P1—S1	109.51 (14)	C35—C36—H36	119.5
O1—P1—S2	114.15 (13)	C31—C36—H36	119.5
C1—P1—S2	111.17 (14)	C46—C41—C42	118.7 (4)
S1—P1—S2	99.14 (6)	C46—C41—P3	123.4 (3)
C11—P2—C31	103.93 (18)	C42—C41—P3	117.9 (3)
C11—P2—C21	102.26 (19)	C43—C42—C41	120.7 (4)
C31—P2—C21	98.52 (18)	C43—C42—H42	119.7
C11—P2—Ru1	116.26 (13)	C41—C42—H42	119.7
C31—P2—Ru1	119.75 (13)	C42—C43—C44	120.1 (4)
C21—P2—Ru1	113.37 (13)	C42—C43—H43	120.0
C61—P3—C51	104.19 (19)	C44—C43—H43	120.0
C61—P3—C41	98.26 (18)	C45—C44—C43	119.7 (4)
C51—P3—C41	102.50 (18)	C45—C44—H44	120.1
C61—P3—Ru1	121.33 (14)	C43—C44—H44	120.1
C51—P3—Ru1	113.93 (14)	C44—C45—C46	120.2 (5)
C41—P3—Ru1	114.01 (13)	C44—C45—H45	119.9
C2—C1—C5	107.2 (4)	C46—C45—H45	119.9
C2—C1—P1	129.1 (3)	C41—C46—C45	120.6 (4)
C5—C1—P1	123.5 (3)	C41—C46—H46	119.7
C2—C1—Fe1	69.4 (3)	C45—C46—H46	119.7
C5—C1—Fe1	69.3 (2)	C56—C51—C52	118.5 (4)
P1—C1—Fe1	123.3 (2)	C56—C51—P3	123.2 (3)
C1—C2—C3	107.9 (4)	C52—C51—P3	118.3 (3)
C1—C2—Fe1	69.8 (3)	C53—C52—C51	120.7 (4)
C3—C2—Fe1	69.8 (3)	C53—C52—H52	119.7
C1—C2—H2	126.0	C51—C52—H52	119.7
C3—C2—H2	126.0	C54—C53—C52	120.4 (5)
Fe1—C2—H2	125.9	C54—C53—H53	119.8
C4—C3—C2	108.7 (4)	C52—C53—H53	119.8
C4—C3—Fe1	69.9 (3)	C53—C54—C55	120.2 (4)
C2—C3—Fe1	69.3 (3)	C53—C54—H54	119.9
C4—C3—H3	125.6	C55—C54—H54	119.9
C2—C3—H3	125.6	C54—C55—C56	119.9 (5)
Fe1—C3—H3	126.8	C54—C55—H55	120.1
C5—C4—C3	107.8 (4)	C56—C55—H55	120.1
C5—C4—Fe1	69.5 (3)	C51—C56—C55	120.3 (5)
C3—C4—Fe1	69.6 (3)	C51—C56—H56	119.8
C5—C4—H4	126.1	C55—C56—H56	119.8
C3—C4—H4	126.1	C62—C61—C66	118.8 (4)
Fe1—C4—H4	126.4	C62—C61—P3	120.2 (3)
C4—C5—C1	108.4 (4)	C66—C61—P3	120.9 (3)
C4—C5—Fe1	70.0 (3)	C61—C62—C63	120.0 (4)
C1—C5—Fe1	69.3 (2)	C61—C62—H62	120.0
C4—C5—H5	125.8	C63—C62—H62	120.0
C1—C5—H5	125.8	C64—C63—C62	120.5 (5)
Fe1—C5—H5	126.5	C64—C63—H63	119.8
C7—C6—C10	107.9 (9)	C62—C63—H63	119.8
C7—C6—Fe1	70.3 (5)	C63—C64—C65	120.3 (5)
C10—C6—Fe1	70.9 (5)	C63—C64—H64	119.8
C7—C6—H6	126.0	C65—C64—H64	119.8
C10—C6—H6	126.0	C64—C65—C66	120.0 (5)
Fe1—C6—H6	124.4	C64—C65—H65	120.0
C6—C7—C8	111.4 (8)	C66—C65—H65	120.0
C6—C7—Fe1	70.9 (4)	C65—C66—C61	120.3 (5)
C8—C7—Fe1	70.8 (4)	C65—C66—H66	119.8
C6—C7—H7	124.3	C61—C66—H66	119.8
C8—C7—H7	124.3	Cl1S—C1S—Cl2S	119.8 (8)
Fe1—C7—H7	125.5	Cl1S—C1S—H1S1	107.4
C7—C8—C9	108.1 (8)	Cl2S—C1S—H1S1	107.4
C7—C8—Fe1	69.9 (4)	Cl1S—C1S—H1S2	107.4
C9—C8—Fe1	70.0 (4)	Cl2S—C1S—H1S2	107.4
C7—C8—H8	125.9	H1S1—C1S—H1S2	106.9

### Hydrogen-bond geometry (Å, º)

**Table:**

*D*—H···*A*	*D*—H	H···*A*	*D*···*A*	*D*—H···*A*
O3—H1*S*···O1^i^	0.82 (1)	1.72 (2)	2.515 (4)	162 (4)
O3—H2*S*···O3^i^	0.82 (1)	2.52 (6)	2.980 (6)	117 (5)

Symmetry code: (i) −*x*+1, −*y*+2, −*z*+1.
